# Ethyl 7-(4-bromo­phen­yl)-5-trifluoro­methyl-4,7-dihydro­tetra­zolo[1,5-*a*]pyrimidine-6-carboxyl­ate

**DOI:** 10.1107/S1600536810033842

**Published:** 2010-08-28

**Authors:** Shi-De Shen, Xiao-Dong Feng, Wei-Hua Yang, Chang-Sheng Yao

**Affiliations:** a Xuzhou Institute of Architectural Technology, Xuzhou 221116, People’s Republic of China; b School of Chemistry and Chemical Engineering, Xuzhou Normal University, Xuzhou 221116, People’s Republic of China; c Key Laboratory of Biotechnology for Medicinal Plants, Xuzhou Normal University, Xuzhou 221116, People’s Republic of China

## Abstract

In the title compound, C_14_H_11_BrF_3_N_5_O_2_, the pyrimidine ring adopts a flattened envelope conformation with *sp* ^3^-hybridized carbon as the flap [deviation = 0.177 (3) Å]. The dihedral angle between tetra­zole and bromo­phenyl rings is 84.3 (1)°. In the crystal, mol­ecules are linked into centrosymmetric dimers by pairs of N—H⋯N hydrogen bonds.

## Related literature

For the biological activity of tetra­zolopyrimidine derivatives, see: Von Nussbaum *et al.* (2010 ▶); Abelman *et al.* (2009 ▶); Dougherty *et al.* (2007 ▶). For ring puckering parameters, see: Cremer & Pople (1975 ▶). For the synthesis, see: Pryadeina *et al.* (2004 ▶).
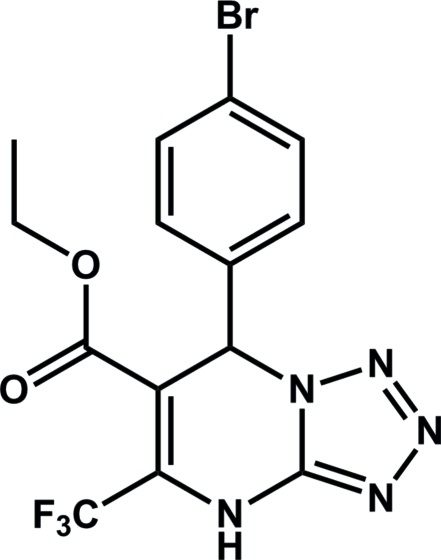


## Experimental

### 

#### Crystal data

C_14_H_11_BrF_3_N_5_O_2_
*M* *_r_* = 418.19
Monoclinic, 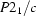

*a* = 18.773 (2) Å
*b* = 10.4716 (11) Å
*c* = 7.8700 (8) Å
β = 92.27 (3)°
*V* = 1545.9 (3) Å^3^
*Z* = 4
Mo *K*α radiation
μ = 2.71 mm^−1^
*T* = 113 K
0.32 × 0.28 × 0.18 mm

#### Data collection

Rigaku Saturn diffractometer
Absorption correction: multi-scan (*CrystalClear*; Rigaku/MSC, 2002 ▶) *T*_min_ = 0.477, *T*_max_ = 0.641
18785 measured reflections
3681 independent reflections
2547 reflections with *I* > 2σ(*I*)
*R*_int_ = 0.065

#### Refinement

*R*[*F* ^2^ > 2σ(*F* ^2^)] = 0.030
*wR*(*F* ^2^) = 0.071
*S* = 0.94
3681 reflections
231 parameters
H atoms treated by a mixture of independent and constrained refinement
Δρ_max_ = 0.43 e Å^−3^
Δρ_min_ = −0.82 e Å^−3^

### 

Data collection: *CrystalClear* (Rigaku/MSC, 2002 ▶); cell refinement: *CrystalClear*; data reduction: *CrystalClear*; program(s) used to solve structure: *SHELXS97* (Sheldrick, 2008 ▶); program(s) used to refine structure: *SHELXL97* (Sheldrick, 2008 ▶); molecular graphics: *SHELXTL* (Sheldrick, 2008 ▶); software used to prepare material for publication: *SHELXL97*.

## Supplementary Material

Crystal structure: contains datablocks I, global. DOI: 10.1107/S1600536810033842/ci5159sup1.cif

Structure factors: contains datablocks I. DOI: 10.1107/S1600536810033842/ci5159Isup2.hkl

Additional supplementary materials:  crystallographic information; 3D view; checkCIF report

## Figures and Tables

**Table 1:** Hydrogen-bond geometry (Å, °)

*D*—H⋯*A*	*D*—H	H⋯*A*	*D*⋯*A*	*D*—H⋯*A*
N1—H1⋯N5^i^	0.83 (2)	2.06 (2)	2.862 (2)	163 (2)

Symmetry code: (i) 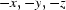.

**Fig. 1.**
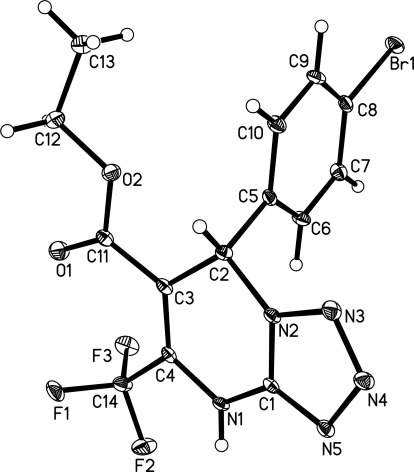
The molecular structure of the title compound, showing 30% probability displacement ellipsoids and the atom-numbering scheme.

**Fig. 2.**
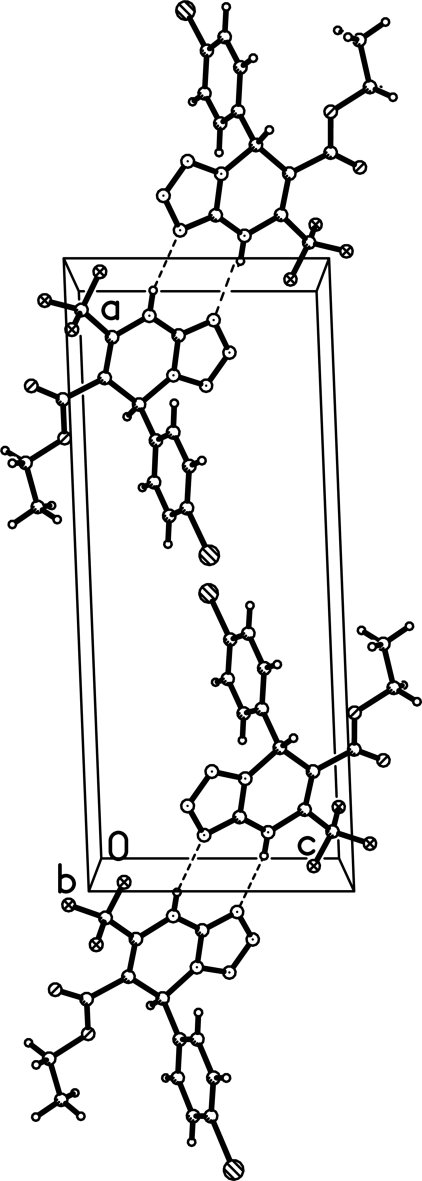
A packing diagram of the title compound. Intermolecular hydrogen bonds are shown as dashed lines.

## supplementary crystallographic information

### Comment

The tetrazolo[1,5-*a*]pyrimidine core represents an interesting pharmacophore with the feature of biological and pharmacological properties, which has human neutrophil elastase inhibitory (Von Nussbaum *et al.*, 2010), late sodium channel blocker (Abelman *et al.*, 2009) and hepatitis B virus surface antigen secretion inhibitory  activities (Dougherty *et al.*, 2007). This led us to pay much attention to the synthesis and bioactivity of compounds containing these two significant fragments. During the synthesis of trifluoromethylated tetrazolo[1,5-*a*]pyrimidine derivatives, the title compound was isolated and its structure was determined by X-ray analysis. The results are presented here.

In the title molecule (Fig.1), the tetrahydropyrimidine ring is in a flattened envelope conformation, with Cremer and Pople (1975) puckering parameters Q, θ, φ of 0.125 (2) Å, 109.7 (9)° and 11.7 (9)°, respectively; atom C2 deviates from the N1/N2/C1/C3/C4 plane (r.m.s. deviation 0.018 Å) by 0.177 (3) Å. The dihedral angle between N1/N2/C1/C3/C4 and C5-C10 planes [89.53 (3)°] shows that they are nearly perpendicular.

The crystal packing is stabilized by intermolecular  N—H···N hydrogen bonds (Table 1 and Fig.2).

### Experimental

The title compound was synthesized according the procedure reported by Pryadeina *et al.*(2004). A mixture of ethyl 4,4,4-trifluoro-3-oxobutanoate (0.01 mol), 4-bromobenzaldehyde (0.01 mol) and 5-aminotetrazole (0.01 mol) in ethanol (20 ml) containing a catalytic amount of hydrochloric acid was heated for 12 h under reflux. Then the solvent was removed under reduced pressure. The residue was added to a solution of p-toluenesulfonic acid (0.05 g) in 100 mL of benzene, and the mixture was heated for 8 h with simultaneous removal of water as azeotrope with benzene. The solution was filtered while hot, the filtrate was evaporated, and the precipitate was recrystallized from ethanol. Cooling the ethanol solution slowly gave single crystals suitable for X-ray diffraction.

### Refinement

The N-bound H atom was located in a difference map and was refined freely [refined N–H length, 0.83 (2)Å]. All other H atoms were placed in calculated positions [C–H = 0.95–1.00 Å] and included in the final cycles of refinement using a riding model, with U_iso_(H) = 1.2U_eq_(parent atom).

### Crystal data

**Table:**

C_14_H_11_BrF_3_N_5_O_2_	*F*(000) = 832
*M**_r_* = 418.19	*D*_x_ = 1.797 Mg m^−^^3^
Monoclinic, *P*2_1_/*c*	Mo *K*α radiation, λ = 0.71073 Å
Hall symbol: -P 2ybc	Cell parameters from 4823 reflections
*a* = 18.773 (2) Å	θ = 2.2–27.9°
*b* = 10.4716 (11) Å	µ = 2.71 mm^−^^1^
*c* = 7.8700 (8) Å	*T* = 113 K
β = 92.27 (3)°	Block, colourless
*V* = 1545.9 (3) Å^3^	0.32 × 0.28 × 0.18 mm
*Z* = 4	

### Data collection

**Table:**

Rigaku Saturn diffractometer	3681 independent reflections
Radiation source: rotating anode	2547 reflections with *I* > 2σ(*I*)
confocal	*R*_int_ = 0.065
Detector resolution: 7.31 pixels mm^-1^	θ_max_ = 27.9°, θ_min_ = 2.2°
ω scans	*h* = −24→24
Absorption correction: multi-scan (*CrystalClear*; Rigaku/MSC, 2002)	*k* = −13→13
*T*_min_ = 0.477, *T*_max_ = 0.641	*l* = −10→10
18785 measured reflections	

### Refinement

**Table:**

Refinement on *F*^2^	Primary atom site location: structure-invariant direct methods
Least-squares matrix: full	Secondary atom site location: difference Fourier map
*R*[*F*^2^ > 2σ(*F*^2^)] = 0.030	Hydrogen site location: inferred from neighbouring sites
*wR*(*F*^2^) = 0.071	H atoms treated by a mixture of independent and constrained refinement
*S* = 0.94	*w* = 1/[σ^2^(*F*_o_^2^) + (0.0321*P*)^2^] where *P* = (*F*_o_^2^ + 2*F*_c_^2^)/3
3681 reflections	(Δ/σ)_max_ = 0.001
231 parameters	Δρ_max_ = 0.43 e Å^−^^3^
0 restraints	Δρ_min_ = −0.82 e Å^−^^3^

### Special details

**Table:**

Geometry. All esds (except the esd in the dihedral angle between two l.s. planes) are estimated using the full covariance matrix. The cell esds are taken into account individually in the estimation of esds in distances, angles and torsion angles; correlations between esds in cell parameters are only used when they are defined by crystal symmetry. An approximate (isotropic) treatment of cell esds is used for estimating esds involving l.s. planes.
Refinement. Refinement of F^2^ against ALL reflections. The weighted R-factor wR and goodness of fit S are based on F^2^, conventional R-factors R are based on F, with F set to zero for negative F^2^. The threshold expression of F^2^ > 2sigma(F^2^) is used only for calculating R-factors(gt) etc. and is not relevant to the choice of reflections for refinement. R-factors based on F^2^ are statistically about twice as large as those based on F, and R- factors based on ALL data will be even larger.

### Fractional atomic coordinates and isotropic or equivalent isotropic displacement parameters (Å^2^)

**Table:**

	*x*	*y*	*z*	*U*_iso_*/*U*_eq_	
Br1	0.468171 (12)	0.24266 (2)	−0.00430 (3)	0.02485 (8)	
F1	0.04617 (6)	0.09175 (12)	0.60550 (15)	0.0283 (3)	
F2	0.00359 (6)	0.18117 (13)	0.37917 (16)	0.0312 (3)	
F3	0.10127 (7)	0.25030 (10)	0.49390 (17)	0.0279 (3)	
H1	0.0275 (12)	0.055 (2)	0.176 (3)	0.033 (7)*	
N1	0.06837 (9)	0.02879 (16)	0.1959 (2)	0.0142 (4)	
N2	0.16300 (9)	−0.09820 (15)	0.1113 (2)	0.0139 (4)	
N3	0.17768 (9)	−0.17880 (16)	−0.0190 (2)	0.0195 (4)	
N4	0.12129 (9)	−0.17855 (17)	−0.1192 (2)	0.0196 (4)	
N5	0.06960 (9)	−0.09971 (15)	−0.0596 (2)	0.0163 (4)	
O1	0.18713 (8)	0.08260 (14)	0.67228 (18)	0.0266 (4)	
O2	0.27052 (7)	−0.04022 (14)	0.55633 (17)	0.0209 (3)	
C1	0.09759 (10)	−0.05253 (18)	0.0835 (2)	0.0134 (4)	
C2	0.21318 (10)	−0.06715 (18)	0.2535 (2)	0.0143 (4)	
H2	0.2315	−0.1483	0.3062	0.017*	
C3	0.17113 (10)	0.00649 (18)	0.3842 (2)	0.0147 (4)	
C4	0.10521 (11)	0.05403 (18)	0.3465 (2)	0.0141 (4)	
C5	0.27570 (10)	0.00933 (19)	0.1898 (2)	0.0143 (4)	
C6	0.26514 (11)	0.12937 (19)	0.1182 (3)	0.0185 (5)	
H6	0.2184	0.1642	0.1091	0.022*	
C7	0.32231 (11)	0.1991 (2)	0.0599 (3)	0.0187 (5)	
H7	0.3150	0.2809	0.0097	0.022*	
C8	0.39009 (11)	0.14732 (19)	0.0761 (2)	0.0172 (5)	
C9	0.40180 (11)	0.02870 (19)	0.1482 (3)	0.0196 (5)	
H9	0.4487	−0.0054	0.1587	0.024*	
C10	0.34446 (11)	−0.03993 (19)	0.2049 (3)	0.0185 (5)	
H10	0.3521	−0.1216	0.2548	0.022*	
C11	0.20751 (11)	0.02294 (19)	0.5533 (3)	0.0174 (4)	
C12	0.31205 (11)	−0.0374 (2)	0.7163 (3)	0.0251 (5)	
H12A	0.2901	−0.0933	0.8012	0.030*	
H12B	0.3145	0.0506	0.7620	0.030*	
C13	0.38555 (12)	−0.0847 (2)	0.6780 (3)	0.0290 (6)	
H13A	0.3820	−0.1701	0.6276	0.043*	
H13B	0.4150	−0.0884	0.7835	0.043*	
H13C	0.4074	−0.0262	0.5979	0.043*	
C14	0.06429 (11)	0.1435 (2)	0.4593 (3)	0.0205 (5)	

### Atomic displacement parameters (Å^2^)

**Table:**

	*U*^11^	*U*^22^	*U*^33^	*U*^12^	*U*^13^	*U*^23^
Br1	0.01555 (14)	0.03165 (14)	0.02782 (14)	−0.00341 (9)	0.00700 (9)	0.00705 (10)
F1	0.0232 (8)	0.0413 (8)	0.0212 (7)	−0.0037 (6)	0.0114 (6)	−0.0076 (6)
F2	0.0184 (7)	0.0410 (8)	0.0338 (8)	0.0131 (6)	−0.0035 (6)	−0.0185 (7)
F3	0.0254 (8)	0.0205 (7)	0.0380 (8)	−0.0022 (5)	0.0034 (6)	−0.0136 (6)
N1	0.0094 (10)	0.0162 (9)	0.0174 (9)	0.0012 (7)	0.0031 (7)	−0.0026 (7)
N2	0.0110 (9)	0.0147 (8)	0.0163 (9)	0.0004 (7)	0.0036 (7)	−0.0035 (7)
N3	0.0184 (10)	0.0208 (10)	0.0194 (9)	0.0007 (8)	0.0033 (8)	−0.0074 (8)
N4	0.0152 (10)	0.0220 (10)	0.0218 (10)	0.0018 (8)	0.0027 (8)	−0.0063 (8)
N5	0.0145 (10)	0.0169 (9)	0.0178 (9)	0.0003 (7)	0.0045 (7)	−0.0036 (7)
O1	0.0224 (9)	0.0357 (9)	0.0217 (8)	0.0044 (7)	0.0022 (7)	−0.0102 (7)
O2	0.0175 (8)	0.0271 (8)	0.0178 (8)	0.0040 (6)	−0.0011 (6)	−0.0042 (6)
C1	0.0111 (11)	0.0130 (9)	0.0163 (10)	−0.0016 (8)	0.0050 (8)	0.0019 (8)
C2	0.0122 (11)	0.0140 (10)	0.0167 (10)	0.0005 (8)	0.0012 (8)	−0.0019 (8)
C3	0.0132 (11)	0.0136 (9)	0.0176 (10)	−0.0029 (8)	0.0051 (8)	−0.0018 (8)
C4	0.0137 (11)	0.0135 (10)	0.0154 (10)	−0.0030 (8)	0.0058 (8)	−0.0013 (8)
C5	0.0137 (11)	0.0164 (10)	0.0130 (10)	−0.0005 (8)	0.0038 (8)	−0.0029 (8)
C6	0.0124 (12)	0.0203 (11)	0.0230 (11)	0.0033 (9)	0.0031 (9)	0.0007 (9)
C7	0.0197 (12)	0.0163 (10)	0.0204 (11)	0.0023 (9)	0.0038 (9)	0.0013 (9)
C8	0.0152 (12)	0.0216 (11)	0.0153 (10)	−0.0036 (9)	0.0052 (8)	−0.0008 (9)
C9	0.0107 (11)	0.0248 (12)	0.0236 (12)	0.0045 (9)	0.0037 (9)	0.0011 (9)
C10	0.0168 (12)	0.0170 (10)	0.0220 (11)	0.0036 (9)	0.0047 (9)	0.0015 (9)
C11	0.0148 (12)	0.0175 (10)	0.0203 (11)	−0.0032 (9)	0.0037 (9)	0.0003 (9)
C12	0.0193 (13)	0.0366 (14)	0.0192 (11)	0.0028 (10)	−0.0031 (9)	0.0003 (10)
C13	0.0206 (13)	0.0371 (14)	0.0292 (13)	0.0054 (11)	−0.0004 (10)	0.0039 (11)
C14	0.0142 (12)	0.0238 (11)	0.0235 (12)	0.0006 (9)	0.0014 (9)	−0.0069 (10)

### Geometric parameters (Å, °)

**Table:**

Br1—C8	1.9025 (19)	C3—C4	1.356 (3)
F1—C14	1.328 (2)	C3—C11	1.482 (3)
F2—C14	1.340 (2)	C4—C14	1.520 (3)
F3—C14	1.338 (2)	C5—C6	1.388 (3)
N1—C1	1.359 (2)	C5—C10	1.391 (3)
N1—C4	1.374 (3)	C6—C7	1.392 (3)
N1—H1	0.83 (2)	C6—H6	0.95
N2—C1	1.328 (2)	C7—C8	1.385 (3)
N2—N3	1.365 (2)	C7—H7	0.95
N2—C2	1.470 (2)	C8—C9	1.379 (3)
N3—N4	1.295 (2)	C9—C10	1.383 (3)
N4—N5	1.371 (2)	C9—H9	0.95
N5—C1	1.320 (2)	C10—H10	0.95
O1—C11	1.201 (2)	C12—C13	1.508 (3)
O2—C11	1.355 (2)	C12—H12A	0.99
O2—C12	1.455 (2)	C12—H12B	0.99
C2—C5	1.522 (2)	C13—H13A	0.98
C2—C3	1.530 (3)	C13—H13B	0.98
C2—H2	1.00	C13—H13C	0.98
			
C1—N1—C4	118.68 (17)	C8—C7—H7	120.6
C1—N1—H1	119.1 (17)	C6—C7—H7	120.6
C4—N1—H1	121.9 (16)	C9—C8—C7	121.45 (19)
C1—N2—N3	108.16 (16)	C9—C8—Br1	119.80 (15)
C1—N2—C2	127.32 (16)	C7—C8—Br1	118.75 (15)
N3—N2—C2	124.50 (16)	C8—C9—C10	119.16 (19)
N4—N3—N2	105.79 (16)	C8—C9—H9	120.4
N3—N4—N5	111.43 (16)	C10—C9—H9	120.4
C1—N5—N4	104.74 (16)	C9—C10—C5	120.74 (19)
C11—O2—C12	116.29 (15)	C9—C10—H10	119.6
N5—C1—N2	109.88 (17)	C5—C10—H10	119.6
N5—C1—N1	129.27 (18)	O1—C11—O2	123.05 (19)
N2—C1—N1	120.84 (18)	O1—C11—C3	127.68 (19)
N2—C2—C5	110.17 (15)	O2—C11—C3	109.27 (17)
N2—C2—C3	106.92 (15)	O2—C12—C13	106.47 (17)
C5—C2—C3	112.42 (16)	O2—C12—H12A	110.4
N2—C2—H2	109.1	C13—C12—H12A	110.4
C5—C2—H2	109.1	O2—C12—H12B	110.4
C3—C2—H2	109.1	C13—C12—H12B	110.4
C4—C3—C11	122.55 (18)	H12A—C12—H12B	108.6
C4—C3—C2	121.92 (18)	C12—C13—H13A	109.5
C11—C3—C2	115.53 (17)	C12—C13—H13B	109.5
C3—C4—N1	122.81 (17)	H13A—C13—H13B	109.5
C3—C4—C14	125.21 (18)	C12—C13—H13C	109.5
N1—C4—C14	111.95 (17)	H13A—C13—H13C	109.5
C6—C5—C10	119.19 (18)	H13B—C13—H13C	109.5
C6—C5—C2	120.69 (18)	F1—C14—F3	108.26 (17)
C10—C5—C2	120.11 (18)	F1—C14—F2	106.58 (17)
C5—C6—C7	120.64 (19)	F3—C14—F2	105.95 (17)
C5—C6—H6	119.7	F1—C14—C4	114.01 (17)
C7—C6—H6	119.7	F3—C14—C4	111.34 (17)
C8—C7—C6	118.80 (19)	F2—C14—C4	110.28 (17)
			
C1—N2—N3—N4	0.1 (2)	C3—C2—C5—C6	−55.7 (2)
C2—N2—N3—N4	−178.49 (17)	N2—C2—C5—C10	−117.49 (19)
N2—N3—N4—N5	0.2 (2)	C3—C2—C5—C10	123.4 (2)
N3—N4—N5—C1	−0.4 (2)	C10—C5—C6—C7	1.0 (3)
N4—N5—C1—N2	0.4 (2)	C2—C5—C6—C7	−179.92 (18)
N4—N5—C1—N1	−178.44 (19)	C5—C6—C7—C8	−0.7 (3)
N3—N2—C1—N5	−0.3 (2)	C6—C7—C8—C9	0.2 (3)
C2—N2—C1—N5	178.17 (17)	C6—C7—C8—Br1	179.84 (15)
N3—N2—C1—N1	178.65 (16)	C7—C8—C9—C10	0.2 (3)
C2—N2—C1—N1	−2.8 (3)	Br1—C8—C9—C10	−179.52 (15)
C4—N1—C1—N5	172.99 (19)	C8—C9—C10—C5	0.1 (3)
C4—N1—C1—N2	−5.8 (3)	C6—C5—C10—C9	−0.7 (3)
C1—N2—C2—C5	−111.0 (2)	C2—C5—C10—C9	−179.76 (18)
N3—N2—C2—C5	67.3 (2)	C12—O2—C11—O1	2.7 (3)
C1—N2—C2—C3	11.5 (2)	C12—O2—C11—C3	−177.93 (16)
N3—N2—C2—C3	−170.26 (16)	C4—C3—C11—O1	−4.0 (3)
N2—C2—C3—C4	−13.1 (2)	C2—C3—C11—O1	175.58 (19)
C5—C2—C3—C4	108.0 (2)	C4—C3—C11—O2	176.71 (17)
N2—C2—C3—C11	167.39 (16)	C2—C3—C11—O2	−3.7 (2)
C5—C2—C3—C11	−71.6 (2)	C11—O2—C12—C13	−166.71 (17)
C11—C3—C4—N1	−173.55 (17)	C3—C4—C14—F1	−65.4 (3)
C2—C3—C4—N1	6.9 (3)	N1—C4—C14—F1	116.58 (19)
C11—C3—C4—C14	8.6 (3)	C3—C4—C14—F3	57.5 (3)
C2—C3—C4—C14	−170.92 (18)	N1—C4—C14—F3	−120.58 (19)
C1—N1—C4—C3	3.5 (3)	C3—C4—C14—F2	174.78 (18)
C1—N1—C4—C14	−178.41 (17)	N1—C4—C14—F2	−3.3 (2)
N2—C2—C5—C6	63.4 (2)		

### Hydrogen-bond geometry (Å, °)

**Table:**

*D*—H···*A*	*D*—H	H···*A*	*D*···*A*	*D*—H···*A*
N1—H1···N5^i^	0.83 (2)	2.06 (2)	2.862 (2)	163 (2)

Symmetry codes: (i) −*x*, −*y*, −*z*.
